# Artificial light at night decreases leaf herbivory in typical urban areas

**DOI:** 10.3389/fpls.2024.1392262

**Published:** 2024-08-05

**Authors:** Yu Cao, Shuang Zhang, Ke-Ming Ma

**Affiliations:** ^1^ State Key Laboratory of Urban and Regional Ecology, Research Center for Eco-Environmental Sciences, Chinese Academy of Sciences, Beijing, China; ^2^ University of Chinese Academy of Sciences, Beijing, China

**Keywords:** artificial light at night, plant functional traits, herbivory, energy flow, urban plant, urban ecosystems

## Abstract

Artificial light at night (ALAN) is exerting growing pressure on natural ecosystems, but its impact on biological interactions remains unclear. This study aimed to assess how ALAN influences leaf functional traits and herbivory in two prevalent street tree species (*Styphnolobium japonicum* (L.) Schott and *Fraxinus pennsylvanica*) through field surveys and paired experiments in the urban areas of Beijing, China. We found that ALAN led to increased leaf toughness and decreased levels of leaf herbivory. Additionally, ALAN showed species-specific effects on leaf nutrients, size as well as defense substances. The findings illustrate that ALAN can significantly alter some key functional traits and ecological processes (nutrient cycling, energy flow). In general, we suggest that high ALAN intensity will be detrimental to the energy flow from urban plants to higher trophic levels, posing a potential threat to the maintenance of biodiversity (e.g., arthropod diversity, bird diversity) in urban ecosystems.

## 1 Introduction

More than 80% of the world’s population lives with daily light pollution, experiencing at least an 8% increase in nighttime brightness compared to natural levels (Gaston et al., 2015; Falchi et al., 2016). Recognized as a major concern in global change for the 21st century, artificial light at night (ALAN) possesses widespread, distinctive, and rapidly growing pollution characteristics that have the potential to alter natural ecosystems (Svechkina et al., 2020; Sanders et al., 2021), particularly in urban areas (Hopkins et al., 2018). The ecological effects of ALAN are increasingly acknowledged in urban ecosystems, particularly on the two sides of streets (Bennie et al., 2016; La Sorte et al., 2017). There are studies indicating that the intensity of ALAN has exceeded the threshold of plant adaptation, leading to changes in their physiological responses (Singhal et al., 2019; Jägerbrand and Spoelstra, 2023). While the ecological impacts of ALAN on urban plants have been observed for a long time, quantifying such effects empirically has been rare (Bennie et al., 2018). To date, relatively little research has been conducted on the effects of ALAN on key ecological processes such as herbivory, which hampers a comprehensive understanding of the impact of ALAN on urban ecosystems (Garrett et al., 2020).

ALAN has been shown to have effects on various plant traits that are important for biotic interactions and ecological functions (Škvareninová et al., 2017). For example, ALAN has been found to influence leaf toughness and the nutrient content (C/N) of plant leaves (Murphy et al., 2022). However, in a five-year study, ALAN exhibited no effects on leaf carbon and nitrogen content in most grassland species (Anic et al., 2022), suggesting mixed results based on current studies. The prolonged exposure to artificial light may affect leaf size and specific leaf area (SLA) (Hughes and Cockshull, 1966), but the strength and direction of these effects are still controversial (Adams and Langton, 2005). While it is recognized that there are similarities between natural light and artificial light in high-light environments, ALAN typically has a limited spectrum, specific wavelengths and brightness compared to natural light (Thorington, 1985). over-illumination of ALAN in one direction towards the plant for a prolonged period of time can result in the plant failing to adapt. Therefore, it is important to evaluate the responses of plants responses to ALAN in urban ecosystems. It has been shown that small-leaved species tend to occur in higher-light environments (Niinemets and Kull, 1994). However, in Mexican tropical lowland rainforests, leaf size in higher light was significantly larger than in low light (Popma et al., 1992). It is widely recognized that lower light conditions lead to higher SLA (Liu et al., 2016). However, in studies of lianas, shade-tolerant lianas did not respond to light, and light-demanding lianas had higher SLA in high light environments (Cai et al., 2008). In addition, a study revealed differential responses of oak and blueberries to ALAN, with oak exhibiting thicker leaves and enhanced defenses, while blueberries displayed no such variation (Cieraad et al., 2023). It is well known that leaf nutrient content, size, SLA, toughness and so on are important functional traits that have profound effects on many biotic interactions, such as herbivory, predation (Carmona et al., 2011) and ecosystem functions, such as energy flow, decomposition, etc (Rosenfield et al., 2020). It is reasonable to predict that ALAN-induced changes in plant functional traits could have profound effects on related biotic interactions and ecological processes, but our current knowledge about this is quite limited (Giavi et al., 2021).

The effects of ALAN on plant functional traits could have profound influences on the interactions between plants and other organisms. Herbivory, one of the most common biological interactions, serves as a suitable metric to gauge this influence (Grenis and Murphy, 2019). All the functional traits mentioned above could affect the feeding of herbivores on plants. There are evidences showing that herbivory can increase with ALAN (Schroer et al., 2019; Mondy et al., 2021; Cieraad et al., 2023). Conversely, studies have also demonstrated that ALAN can enhance plant chemical defense levels (including the contents of phenolic compounds, terpenes and tannins) and leaf toughness (Chapin et al., 1987; Dudt and Shure, 1994), all of which can impede the herbivory process. Chemicals such as phenolic compounds, terpenes and tannin content could reduce plant palatability and nutritional value (Bennett and Wallsgrove, 1994), which in turn reduces herbivory levels. Leaf with higher toughness can also impede herbivores’ feeding (Salgado-Luarte et al., 2023). We suggest that the ALAN-mediated effect on plants should be consistent with the prediction of the growth-defense hypothesis (Coley et al., 1985), which posits that plants allocate more resources to defense rather than to growth under resource stress. Generally, leaves grown under high ALAN conditions should have higher mechanical toughness and chemical defense levels. Thus, we predicted that under high ALAN conditions, plants would allocate more resources to defend against herbivores. Overall, there are still significant knowledge gaps about the potential mechanisms of ALAN effects on herbivory, to our knowledge, no previous study has evaluated the possible effects. Our study supplemented research on herbivory in complex urban environments by quantifying the effects of ALAN and interactions with plant functional traits on herbivory.

In this study, we assessed the effects of ALAN on plant functional traits and herbivory levels using two of the most prevalent tree species (*Fraxinus pennsylvanicaa* and *Styphnolobium japonicum* (L.) Schott) in urban areas of Beijing, China. These two tree species show significant differences in leaf traits. Compared to *F. pennsylvanica*, *S. japonicum* (L.) Schott possesses smaller and softer leaves, rendering them more attractive to herbivores. Therefore, the two species may respond differently to ALAN, leading to different ecological effects. Specifically, within a typical highly urbanized city in China, we hypothesized that ALAN can influence plant nutrient and defense properties, which in turn influence herbivory levels.

## 2 Materials and methods

### 2.1 Study site

The study was conducted in Beijing, China, situated at a latitude of 39.4–41.6°N and a longitude of 115.7–117.4°E, representing one of the most urbanized areas in China. The study area is approximately 42.4 kilometers from south to north and 20 kilometers from east to west, covering approximately 848.4 square kilometers. Based on the Köppen climate classification, Beijing belongs to temperate continental humid climatic area (Koppen, 1918). During the study period in August 2022, the average high temperature in Beijing was 31°C, the average low temperature was 21°C, and the total precipitation was about 78.8 mm (China Meteorological Network: https://data.cma.cn).

**Figure 1:**
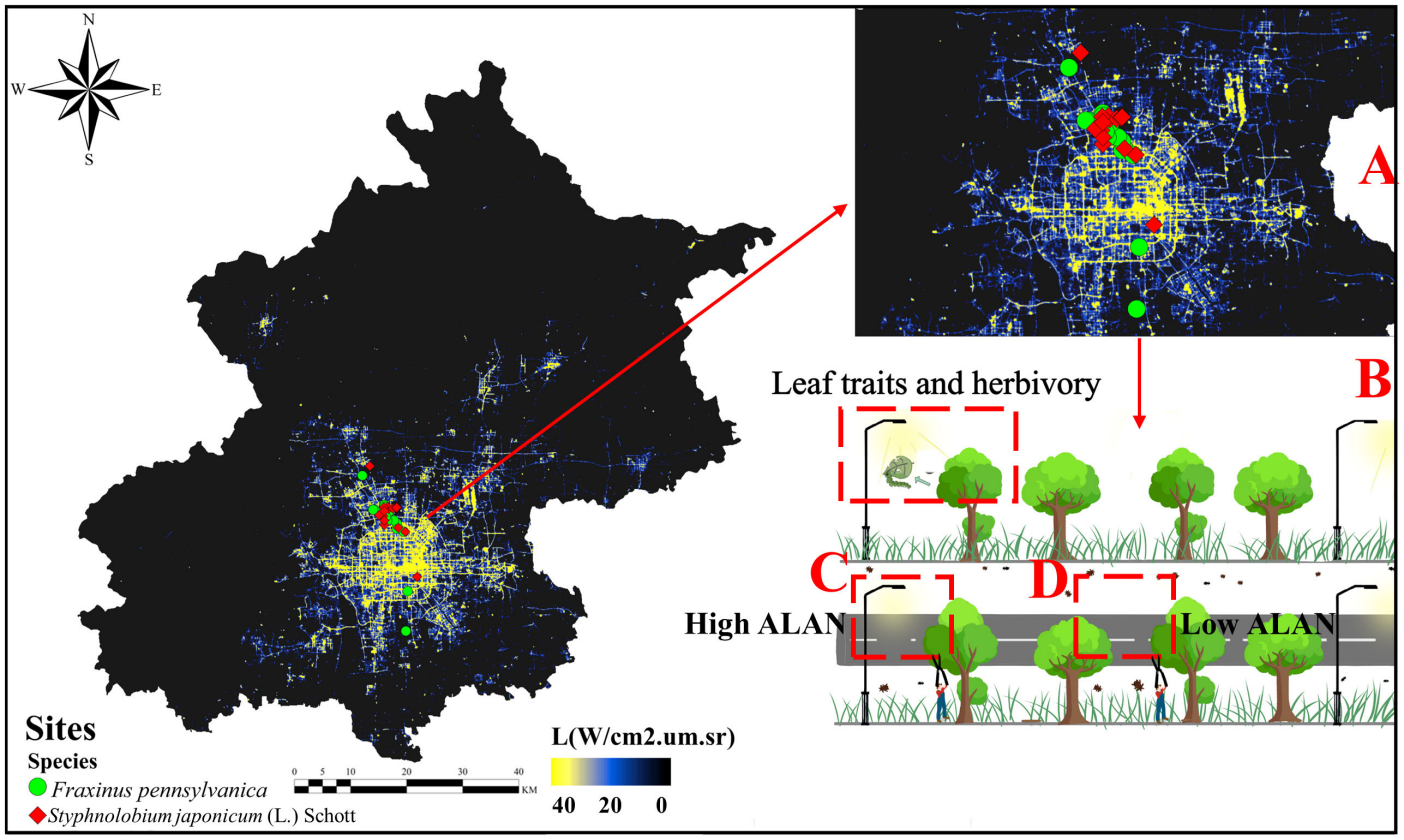
Nighttime light image of the study area in Beijing with a sampling schematic from Luojia 1 (sourced from Hubei Data and Application Center High Resolution Earth Observation System – https://59.175.109.173:8888/app/login.html). **(A)** Distribution of actual sampling points with a resolution of 130m. Red points indicate *Styphnolobium japonicum* (L.) Schott sampling sites, while green points represent *Fraxinus pennsylvanica* sampling sites. **(B)** Schematic illustration of sampling under streetlights. **(C)** Sampling points under high ALAN conditions (directly facing the streetlight and towards the road). **(D)** Sampling points under low ALAN conditions (dark areas between two streetlights that were not directly illuminated by streetlights, towards the road).

In this study, we selected sampling sites along the illuminated road areas of the main axes of Beijing, which are usually illuminated at night (**Figure 1A**) based on satellite illumination data from Luo Jia 1. Considering the accessibility of the area, we selected 30 sampling sites within the determined range, and at each site, we randomly selected the sampled trees. The intensity of illumination at these sites was consistent with the global urban lighting background (Shi et al., 2023). To prevent spatial non-independence among the sites, a minimum distance of 100 meters was maintained between each sampling site (Deb et al., 2013; Li et al., 2018; Koyata et al., 2021). All study sites are illuminated by high-pressure sodium (HPS) lamps, which remain the primary source of light on Beijing’s streets (Beijing Municipal Commission of Urban Management: https://csglw.beijing.gov.cn). According to the “Urban Road Lighting Design Standard (CJJ45–2015)”, the wavelength of urban street high-pressure sodium lamps is generally in the range of 570–600nm, and the CCT is between 3000K and 3500K. Since illuminance (lx), radiation energy of light (W m^-2^) and photon flux density (μmol m^-2^ s^-1^) are multiple measures of light intensity, we chose illuminance as the indicator of ALAN, which is the easiest to understand and obtain. In the summer, street lights in Beijing are typically on from 7:30 p.m. to around 5:00 a.m. the next day (Beijing Municipal Commission of Urban Management: https://csglw.beijing.gov.cn). Within each site, a 30-meter sample strip was established along the roadside (**Figure 1B**), comprising 6 to 7 trees spaced approximately 5 m apart from one another.

### 2.2 Experimental design

The study was designed to investigate the effects of ALAN on leaf nutrient content, defense traits, and herbivory levels of two common urban tree species. *Styphnolobium japonicum* (L.) Schott and *Fraxinus pennsylvanica* are both common street tree species in Beijing. They have similar phenology, both start to grow leaves in April, fruiting during late summer to early fall and in late fall to early winter, the leaves fall off (Westwood et al., 2017; Mackinder and Staniforth, 1997). Both *S. japonicum* (L.) Schott and *F. pennsylvanica* were in the early stages of fruiting during the sampling period. Both of the two species are light-favoring and can tolerate a wide range of urban pollution and soil conditions (Lesica, 2001; Lim, 2014). However, the leaf traits of the two tree species are distinctly different, with *S. japonicum* (L.) Schott’s leaves are smaller and narrower in size, as well as more tender and nutritious than those of *F. pennsylvanica*, which makes *S. japonicum* (L.) Schott ‘s leaves are more desirable to herbivores. Therefore, leaves were collected from *S. japonicum* (L.) Schott and *F. pennsylvanica* trees under high and low ALAN conditions, respectively, for a total of 180 trees across 30 sample sites. Leaf functional traits susceptible to ALAN, including leaf size, tannin content, specific leaf area (SLA), leaf water content (LWC), leaf toughness, leaf carbon (C), nitrogen (N), phosphorus content (P), and C/N ratio, were assessed. Leaf herbivory from insect feeding was also evaluated. Light intensity data of ALAN were obtained by a handheld photometer. The experiments were designed to further understand the ecological consequences of ALAN in urban environments.

### 2.3 Illuminance measurement

At each site, the nighttime illumination was measured utilizing a photometer (CEM DT-1309, Shenzhen Everbest Machinery Industry Co., Ltd. Guangdong, China) with an accuracy of 0.01 lux. During the experimental phase, we controlled the illuminance measurements that occurred around 9:00 pm. Given that we collected leaves at a height of 1.5 to 2 m above the ground, we maintained the measurement height at the same height. The photometer was oriented towards the street road during the light measurement to simulate the real situation of the plant leaves being illuminated by ALAN. The instrument was calibrated to 0 lux before each measurement, and measurements were repeated three times for each light environment.

### 2.4 Collection and measurement of plant leaves

At each site, plant leaf samplings were conducted in pairs under high (mean=13.43 lux, SE=1.16 lux, n=90) and low (mean=1.52 lux, SE=0.18 lux, n=90) ALAN conditions, focusing on the two most prevalent street trees species (*Styphnolobium japonicum* (L.) Schott and *Fraxinus pennsylvanica*) in urban Beijing. In August 2022, within each of the 30 sample sites, we selected three trees subjected to high ALAN conditions (**Figure 1C**) and three trees exposed to low ALAN conditions (**Figure 1D**), culminating in a total of 180 trees. For each tree, 30 leaves were collected from the distal end of external branches at a height of 1.5 to 2 meters above the ground using high branching shears. All collected leaves were fully expanded and at their mature stage of the growth season. Only leaves facing the street road, corresponding to the direction of light measurements were collected, totaling 5,490 leaves. For each leaf, we assessed various leaf functional traits that might be susceptible to ALAN, including leaf size, leaf tannin content, specific leaf area (SLA), leaf water content (LWC), leaf toughness, leaf carbon (C), nitrogen (N), phosphorus content (P) and C/N, as well as herbivory level. In this regard, leaf nitrogen, phosphorus, and water content represent the nutrient value of plant leaves, leaf carbon content, C/N, leaf toughness, and tannins indicate the level of leaf defense, while leaf size and specific leaf area represent the growth strategy of the plant, with larger leaf size and specific leaf area often implying lower inputs into defense (Peschiutta et al., 2018).

Leaf size was quantified with the ImageJ software (https://imagej.net), and the level of leaf herbivory resulting from chewing insects was also assessed with this software (Lowman and Schowalter, 2012). Herbivory was calculated as the proportion of the leaf size consumed by insect feeding, expressed as a percentage: Herbivory = leaf insect eating area/total leaf area × 100% (Kozlov et al., 2020). All visible damages were assumed to be attributable to insect herbivores. Leaf toughness was gauged as the force (measured in Pa) needed to penetrate the leaf with a portable hardness tester (Fruit & Vegetable Tester, Model: GY-2, Wenzhou Weidu Electronics Co., LTD, Zhejiang, China). Leaf water content was determined as LWC (%) = (fresh weight of the leaves - dry weight of the leaves)/fresh weight of the leaves × 100. Fresh leaf weight was measured using a precision electronic balance (AR2140, OHAUS, United States) accurate to 0.001g. Collected leaves were dried in an oven at 55°C for 48 hours until a constant weight and then the carbon, nitrogen and tannin contents were measured.

Samples of 0.1 g were taken and analyzed for carbon and nitrogen content using an elemental analyzer (Vario EL cube, Elementary Analysensysteme GmbH, Germany). The sample was fully combusted and decomposed in a combustion tube at a high temperature of 1150°C. The mixed gas components were separated by reduction and adsorption, and the separated gases were detected with the carrier gas into a thermal conductivity cell detector (TCD). The phosphorus content was determined by H_2_SO_4_-H_2_O_2_ vanadium-molybdenum yellow colorimetric method (Li et al., 2023). The solution to be measured was prepared by decocting 0.1 g of plant sample with H_2_SO_4_-H_2_O_2_ and adding the indicator. The phosphorus content was subsequently obtained by spectrophotometer (UV-1900i, Shimadzu Corporation, Japan) using colorimetric method. The calculation formula was as follows in Equation 1:


(1) $$P\;\left(g/kg\right)=\;\rho \;\times V\times ts\times 10^{-3}/m$$

Where $\rho \;\left(\mu g\;ml^{-1}\right)\;$ is the mass concentration of phosphorus in the colorimetric solution found from the standard curve. *V* (mL) is the volume of the colorimetric solution. *ts* is the splitting multiplier, volume of the decoction solution fixing (mL)/volume of the absorbed decoction solution (mL). *m* (g) is the mass of the dry sample.

Tannin content was determined by the Folin-Denis method (Elgailani et al., 2014). 0.1 g of the treated sample was taken and added to Folin-Denis chromogenic agent, followed by measuring the absorbance of the reaction solution using an ultraviolet spectrophotometer (UV-2450, Shimadzu Corporation, Japan). The calculation formula was as follows in Equation 2:


(2) $$Tannin\;\left(g/kg\right)=\;C\;\times V\times ts\times 10^{-3}/m$$

Where $C\;\left(\mu g\;ml^{-1}\right)\;$ is the mass concentration of tannins in the colorimetric solution found from the standard curve. *V* (mL) is the volume of the colorimetric solution. *ts* is the splitting multiplier, volume of the decoction solution fixing (mL)/volume of the absorbed decoction solution (mL). *m* (g) is the mass of the dry sample.

Each trait was measured at least three times for the 30 leaves collected from each tree. The average value of each tree was used in data analysis.

### 2.5 Statistical analysis

We speculated that there are differences in the responses of the study species to light, so we performed the following analyses in *Styphnolobium japonicum* (L.) Schott and *Fraxinus pennsylvanica* separately. We assessed the effects of ALAN on leaf functional traits (C, N, P, C/N, LWC, Tannin, Leaf toughness, Leaf size, SLA) using linear mixed models with the ‘lme4’ and ‘lmerTest’ packages in R (Bates et al., 2015). In these models, the sample sites were treated as a random effect. To improve the normality of the residuals, P, C, C/N, leaf toughness, leaf size and SLA were log-transformed, and N was square-transformed. Given that herbivory is proportional data and the presence of a zero-inflated beta regression mixed model (with logit link) was employed using the ‘glmmTMB’ package, also with sampling sites as a random effect. Model diagnostics were carried out to examine the normality and homogeneity of the residuals.

To assess the combined effects of ALAN and leaf traits on herbivory, a generalized linear mixed model of beta regression (with logit link and zero inflation) was conducted using the ‘glmmTMB’ package. Sample sites were set as a random effect.

All independent variables were standardized to facilitate the direct comparison of regression coefficients. The model simplification process was executed through the ‘dredge’ function within the ‘MuMIn’ package. Finally, the model with the lowest AICc value was selected as the final model (Burnham et al., 2011). All mixed-effect models passed the collinearity test and all statistical analyses were performed using R version 4.2.2 (R Core Team, 2022).

## 3 Results

### 3.1 The effects of ALAN on leaf traits and herbivory

**Figure 2:**
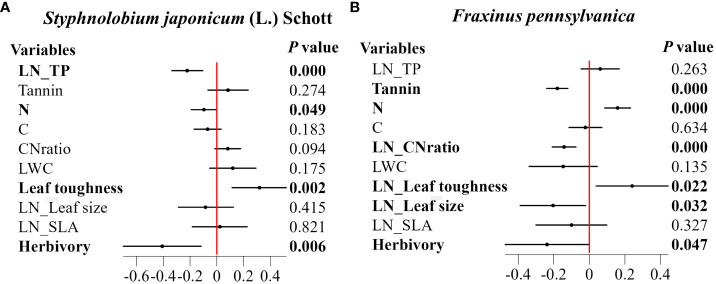
Effect of ALAN on leaf functional traits and herbivory. **(A)** *Styphnolobium japonicum* (L.) Schott. **(B)** *Fraxinus pennsylvanica*. The figure presents standardized regression coefficients along with their 95% confidence intervals (CI) and corresponding *p*-values. Bolded coefficients signify significant effects (*p*< 0.05). Horizontal solid lines represent the 95% confidence intervals, and solid purple squares represent regression coefficients.

The results showed that for both species, ALAN increased leaf toughness while decreasing herbivory. In addition, the zero-inflation part of the model illustrates that the percentage of leaves with no signs of herbivory increased with the intensity of ALAN (0.97 ± 0.32, *P*=0.002 in *Styphnolobium japonicum* (L.) Schott, and -0.71 ± 0.27, *P*=0.009 in *Fraxinus pennsylvanica*). ALAN also showed species-specific effects on other leaf traits (**Figure 2**). In the case of*.S. japonicum* (L.) Schott, ALAN significantly reduced leaf phosphorus (TP) and nitrogen content (N) (**Figure 2A**). For *F. pennsylvanica*, ALAN decreased leaf tannins, C/N and leaf size, but it caused an increase in leaf nitrogen content (N) (**Figure 2B**).

### 3.2 Effects of plant functional traits and their interactions with ALAN on herbivory

**Figure 3:**
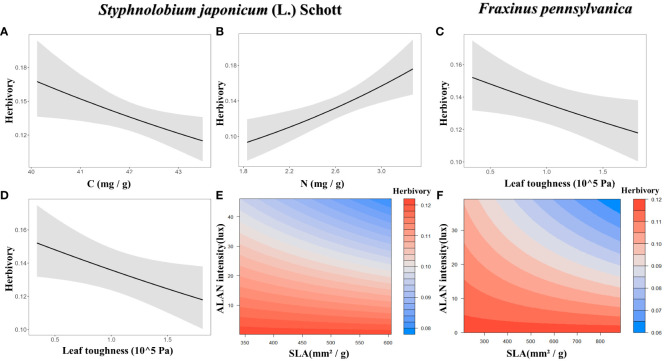
Effects of plant functional traits and their interactions with ALAN on herbivory. Only the optimal model was shown. **(A)** Effect of leaf carbon content under *Styphnolobium japonicum* (L.) Schott. **(B)** Effect of leaf nitrogen content under *S. japonicum* (L.) Schott. **(C)** Effect of leaf toughness under *Fraxinus pennsylvanica*. **(D)** Effect of leaf toughness under *S. japonicum* (L.) Schott. **(E)** Effect of SLA and ALAN interaction under *S. japonicum* (L.) Schott. **(F)** Effect of SLA and ALAN interaction under *F. pennsylvanica*. Non-significant associations were not shown.

**Figure 4:**
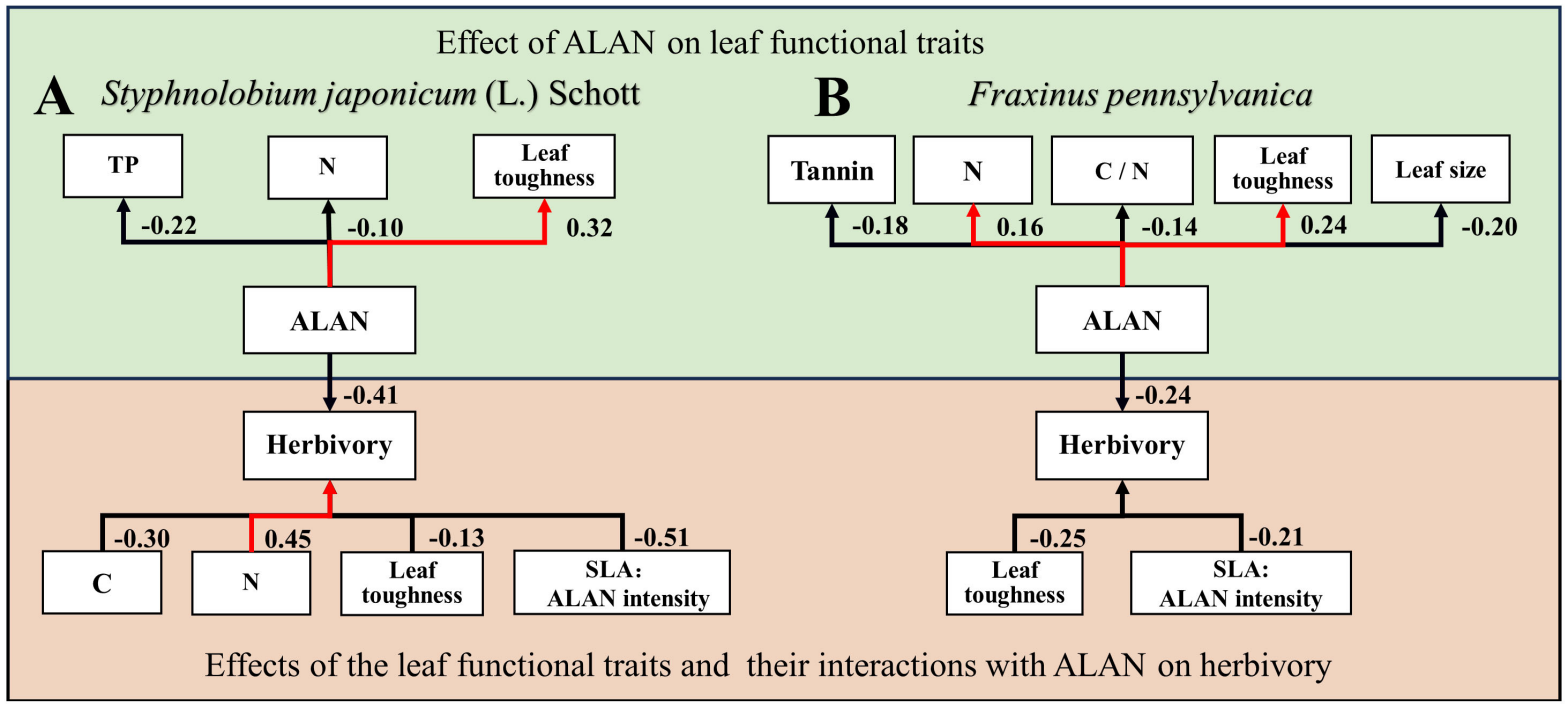
Schematic pathways for the effects of ALAN on plant functional traits and herbivory for **(A)** *Styphnolobium japonicum* (L.) Schott and **(B)** *Fraxinus pennsylvanica*. The green part indicates the effect of ALAN on leaf functional traits, while the orange part indicates the effects of plant functional traits and their interactions with ALAN on herbivory. Red lines signify positive effects, while black lines indicate negative effects. The numbers correspond to standardized regression coefficients, with the direction of the arrow representing the regression relationship.

For *Styphnolobium japonicum* (L.) Schott, herbivory decreased with leaf carbon content (**Figure 3A**) and leaf toughness (**Figure 3D**), while increasing with leaf nitrogen content (**Figure 3B**). Additionally, the interaction between SLA and ALAN showed a significant negative effect on herbivory. The higher the SLA and ALAN, the lower of herbivory (**Figures 3E**, **4A**). For *Fraxinus pennsylvanica*, herbivory decreased with leaf toughness (**Figure 3C**). Meanwhile, we also detected negative interactive effects between SLA and ALAN on herbivory. Herbivory decreased with increasing SLA and ALAN and increased with decreasing SLA and ALAN (**Figures 3F**, **4B**). We integrated the results of the above analyses into a path diagram to intuitively illustrate both the effect paths of ALAN on leaf functional traits and herbivory (**Figure 4**). Notably, ALAN’s influence is more pronounced on *F. pennsylvanica* (**Figure 4B**).

## 4 Discussion

Through a paired design and extensive field investigation, we demonstrated that ALAN can enhance leaf toughness while concurrently reducing leaf herbivory levels in two of the most common street tree species in Beijing, China. Furthermore, ALAN had species-specific effects on leaf nutritional contents and defensive traits. These results provided novel insights into the ecological effects of ALAN in urban ecosystems.

We observed that in both species, ALAN led to higher leaf toughness and lower herbivory levels. This observation indicated that ALAN may profoundly influence some key functional traits and ecological processes related to herbivory and nutrient cycling. Leaf toughness is one of the most crucial leaf traits, representing its structural strength and mechanical defense (Onoda et al., 2011), positively associated with leaf lifespan (Peeters et al., 2007). Leaves grown in higher light conditions might allocate more resources to structural compounds like fiber, contributing to increased leaf toughness (Onoda et al., 2011; Kitajima et al., 2012), which is consistent with previous findings (Murphy et al., 2022; Cieraad et al., 2023). In addition, we found that herbivory declined under high ALAN conditions. This may be related to the influence of leaf defense on herbivore preferences for plants (Cronin and Lodge, 2003). Higher leaf toughness implies intensified mechanical defense, hindering herbivore feeding and resulting in lower herbivory levels (Caldwell et al., 2016). It’s noteworthy that leaves with higher toughness tend to decompose at a slower rate, potentially affecting the nutrient cycling rate negatively (Sena et al., 2020). The decrease in herbivory could also be attributed to the prolonged exposure of herbivores to their predators under ALAN (Herms and Mattson, 1992; Bae et al., 2016), but further studies are needed to confirm this. Additionally, the results of this study are unlikely to be affected by near road pollutants since both the high and low ALAN sampling sites were at the same distance from the source of road pollution. It is no doubt that the possible effect of road pollution on herbivory should be considered in future researches (Furlan et al., 2004). In general, we suggest that high ALAN intensity will be detrimental to the energy flow from plants to higher trophic levels, posing a potential threat to the maintenance of biodiversity (e.g., arthropod diversity, bird diversity) in urban ecosystems.

We observed a negative association between herbivory and the interaction of ALAN and SLA in both tree species. SLA, the ratio of leaf area to leaf dry mass, not only reflects differences in species growth rates (Pérez-Harguindeguy et al., 2013) but also indicates variations in species nutrient investment (Wright et al., 2004). In this study, we found in both *Styphnolobium japonicum* (L.) Schott and *Fraxinus pennsylvanica*, higher SLA and ALAN corresponded to lower herbivory. Conversely, lower SLA and ALAN correlated with higher herbivory. SLA can indirectly reflect the adaptive capacity of plants under different light intensities (McIntyre and Strauss, 2014). Generally, under lower light conditions, plants exhibit higher SLA, resulting in thinner and tastier leaves (Liu et al., 2016). This response helps plants improve light capture efficiency and maximize carbon (Gommers et al., 2013). However, high light conditions can limit the increase in SLA, leading to thicker leaves with reduced light capture efficiency and nutrition, which is less conducive to herbivore feeding (Liu et al., 2016), ultimately resulting in decreased herbivory. We also explored the effects of other plant functional traits on herbivory, but these effects were species-specific. For *S. japonicum* (L.) Schott, herbivory decreased with leaf carbon content and leaf toughness but increased with leaf nitrogen content. Leaf carbon content is a major component in the formation of carbon-based defense compounds (Stiegel et al., 2017), and leaf toughness represents the plant’s mechanical defenses. Therefore, higher leaf carbon and toughness can lead to a decrease in leaf herbivory. On the other hand, higher leaf nitrogen content represents higher leaf nutrition, attracting more herbivores (Meloni et al., 2012), resulting in increased herbivory. Similarly, *F. pennsylvanica* also showed a decrease in herbivory with increased leaf toughness. The reproductive state of plants can interfere with the response of plants to selection pressure mediated by herbivores (Palacio et al., 2021). However, the species in this study were all in their early stages of fruiting during the sampling period, thus the possible influence of different reproductive status on the results has been avoided. We suggest that the role of reproductive status and traits (such as seed size) in mediating the responses of plants to ALAN should be explicitly evaluated in future studies (Palacio et al., 2021; Palacio and Ordano, 2023). In total, ALAN could potentially reshape the feeding preferences of herbivorous insects on leaves. Given that our focus was on herbivory caused by chewing insects, further studies are needed to validate the effects of ALAN on other herbivore groups (e.g., leaf miners, leaf gallers).

We observed that ALAN can affect other plant functional traits, but these effects were species-specific. In *Styphnolobium japonicum* (L.) Schott, ALAN had negative effects on leaf phosphorus and nitrogen content. For *Fraxinus pennsylvanica*, ALAN led to lower tannin contents, C/N and leaf size, and higher leaf nitrogen content. The study demonstrated that responses to ALAN are contingent on the plant species (Kwak et al., 2018). Notably, as we described in the Experimental design of the Method parts, the leaf traits of *S. japonicum* (L.) Schott make them more vulnerable to herbivores. The influence of light on different plant species can lead to varied resource allocation patterns for growth and defense (Muiruri et al., 2019). Consequently, in higher ALAN environments, *S. japonicum* (L.) Schott might allocate more resources to defense, leading to a decrease in nutrient content, such as leaf phosphorus and nitrogen content. In contrast, *F. pennsylvanica*, which has less tasty leaves, might preferentially allocate resources for growth rather than defense. According to the C-N balance hypothesis (Coley et al., 1985), an increase in C/N is positively correlated with the content of chemical defense compounds in leaves. It has long been known that species with larger leaves are more susceptible to the adverse effects caused by intense light exposure (Bragg and Westoby, 2002). In addition, we strictly controlled the orientation, height, position and age of the collected leaves as uniformly as possible to minimize the sub-individual variation of leaf traits (Herrera, 2009). However, there might be sub-individual variation in leaf traits and herbivory in response to ALAN, and future studies need to consider the variation to fully understand the complexity of factors affecting herbivory levels in urban environments. In general, for a comprehensive understanding of the mechanisms and ecological consequences of ALAN on plant traits, more plant species should be included in future studies.

## 5 Conclusion

In conclusion, this study reveals that ALAN exerts a negative effect on herbivory, which is one of the most crucial energy flow pathways in ecosystems. Furthermore, ALAN showed species-specific effects on leaf functional traits and will also affect herbivory through interactions with leaf traits. These results emphasize that high ALAN is detrimental to the flow of energy from plants to higher trophic levels, and further suggest that ALAN may have far-reaching effects on the maintenance of urban biodiversity and ecosystem functioning.

## Data availability statement

The raw data supporting the conclusions of this article will be made available by the authors, without undue reservation.

## Author contributions

YC: Data curation, Formal analysis, Investigation, Resources, Software, Validation, Visualization, Writing – original draft. SZ: Conceptualization, Funding acquisition, Methodology, Project administration, Writing – review & editing. K-MM: Supervision, Writing – review & editing.

## Supplementary material

Supplementary Table 1 Effect of ALAN on leaf functional traits and herbivory of *Styphnolobium japonicum* (L.) Schott. All variables in the table except herbivory were standardized in linear mixed model, and herbivory utilized a generalized linear mixed model. Note. Bold coefficients indicate *p*<.05.

Supplementary Table 2 Effect of ALAN on leaf functional traits and herbivory of *Fraxinus pennsylvanica*. All variables in the table except herbivory were standardized in linear mixed model, and herbivory utilized a generalized linear mixed model. Note. Bold coefficients indicate *p*<.05.

Supplementary Table 3 Effects of plant functional traits on herbivory under *Styphnolobium japonicum* (L.) Schott. Only the optimal linear mixed model was shown. All independent variables in the model are standardized. Note. Bold coefficients indicate *p*<.05.

Supplementary Table 4 Effects of plant functional traits and their interactions with ALAN on herbivory under *Styphnolobium japonicum* (L.) Schott. Only the optimal linear mixed model was shown. All independent variables in the model are standardized. Note. Bold coefficients indicate *p*<.05.

Supplementary Table 5 Effects of plant functional on herbivory under *Fraxinus pennsylvanica*. Only the optimal linear mixed model was shown. All independent variables in the model are standardized. Note. Bold coefficients indicate *p*<.05.

Supplementary Table 6 Effects of plant functional traits and their interactions with ALAN on herbivory under *Fraxinus pennsylvanica*. Only the optimal linear mixed model was shown. All independent variables in the model are standardized. Note. Bold coefficients indicate *p*<.05.
