# Supplementary material for: Artificial light at night decreases leaf herbivory in typical urban areas
Source: Front Plant Sci. 2024 Aug 5;15:1392262. doi: 10.3389/fpls.2024.1392262 (PMC11330841; doi:10.3389/fpls.2024.1392262)
Supplement: Supplementary Table 1 — Effect of ALAN on leaf functional traits and herbivory of Styphnolobium japonicum (L.) Schott. All variables in the table except herbivory were standardized in linear mixed model, and herbivory utilized a generalized linear mixed model. Note. Bold coefficients indicate p<.05. [file Table_1.docx]

Supplementary Material

Supplementary Table 1. Effect of ALAN on leaf functional traits and herbivory of *Styphnolobium japonicum* (L.) Schott. All variables in the table except herbivory were standardized in linear mixed model, and herbivory utilized a generalized linear mixed model.

| **Variables** | **Mean ± SE** | **t-value** | ***p*-value** |
| --- | --- | --- | --- |
| **LN_ TP** | **-0.221 ± 0.006** | **-3.765** | **0.000** |
| Tannin | 0.008 ± 0.008 | 1.102 | 0.274 |
| **N** | **-0.010 ± 0.005** | **-1.999** | **0.049** |
| C | 0.007 ± 0.005 | -1.343 | 0.183 |
| CNratio | 0.008 ± 0.005 | 1.694 | 0.094 |
| LWC | 0.120 ± 0.009 | 1.368 | 0.175 |
| **Leaf toughness** | **0.320 ± 0.102** | **3.131** | **0.002** |
| LN_ Leaf size | -0.008 ± 0.104 | -0.818 | 0.415 |
| LN_SLA | 0.002 ± 0.104 | 0.226 | 0.821 |
| **Herbivory** | **-0.408 ± 0.148** | **-2.75** | **0.006** |

Note. Bold coefficients indicate *p*＜.05.

Supplementary Table 2. Effect of ALAN on leaf functional traits and herbivory of *Fraxinus pennsylvanica*. All variables in the table except herbivory were standardized in linear mixed model, and herbivory utilized a generalized linear mixed model.

| **Variables** | **Mean ± SE** | **t-value** | ***p*-value** |
| --- | --- | --- | --- |
| LN_ TP | 0.006 ± 0.005 | 1.129 | 0.263 |
| **Tannin** | **-0.180 ± 0.003** | **-5.952** | **0.000** |
| **N** | **0.159 ± 0.004** | **4.305** | **0.000** |
| C | -0.002 ± 0.005 | -0.482 | 0.631 |
| **LN_ CNratio** | **-0.142 ± 0.003** | **-4.187** | **0.000** |
| LWC | -0.147 ± 0.010 | -1.508 | 0.135 |
| **LN_ Leaf toughness** | **0.241 ± 0.104** | **2.327** | **0.022** |
| **LN_ Leaf size** | **-0.204 ± 0.009** | **-2.183** | **0.032** |
| LN_SLA | -0.010 ± 0.101 | -0.985 | 0.327 |
| **Herbivory** | **-0.238 ± 0.120** | **-1.986** | **0.047** |

Note. Bold coefficients indicate *p*＜.05.

Supplementary Table 3. Effects of plant functional traits on herbivory under *Styphnolobium japonicum* (L.) Schott. Only the optimal linear mixed model was shown. All independent variables in the model are standardized.

| Variables | Mean ± SE | z-value | *p*-value |
| --- | --- | --- | --- |
| **C** | **-0.128 ± 0.056** | **-2.28** | **0.023** |
| **N** | **0.193 ± 0.059** | **3.29** | **0.001** |
| **Leaf toughness** | **-0.067 ± 0.031** | **-2.12** | **0.034** |

Note. Bold coefficients indicate *p*＜.05.

Supplementary Table 4. Effects of plant functional traits and their interactions with ALAN on herbivory under *Styphnolobium japonicum* (L.) Schott. Only the optimal linear mixed model was shown. All independent variables in the model are standardized.

| Variables | Mean ± SE | z-value | *p*-value |
| --- | --- | --- | --- |
| **SLA * ALAN intensity** | **-0.151 ± 0.038** | **-4.008** | **0.000** |

Note. Bold coefficients indicate *p*＜.05.

Supplementary Table 5. Effects of plant functional on herbivory under *Fraxinus pennsylvanica*. Only the optimal linear mixed model was shown. All independent variables in the model are standardized.

| Variables | Mean ± SE | z-value | *p*-value |
| --- | --- | --- | --- |
| **Leaf toughness** | **-0.086 ± 0.269** | **-3.198** | **0.001** |

Note. Bold coefficients indicate *p*＜.05.

Supplementary Table 6. Effects of plant functional traits and their interactions with ALAN on herbivory under *Fraxinus pennsylvanica*. Only the optimal linear mixed model was shown. All independent variables in the model are standardized.

| Variables | Mean ± SE | z-value | *p*-value |
| --- | --- | --- | --- |
| **SLA * ALAN intensity** | **-0.067 ± 0.028** | **-2.384** | **0.017** |

Note. Bold coefficients indicate *p*＜.05.
